# Symptom palliation of hypofractionated radiotherapy for patients with incurable inflammatory breast cancer

**DOI:** 10.1186/s13014-019-1320-2

**Published:** 2019-06-20

**Authors:** Hoon Sik Choi, Hong Seok Jang, Ki Mun Kang, Byung-ock Choi

**Affiliations:** 1 Department of Radiation Oncology, Gyeongsang National University School of Medicine and Gyeongsang National University Changwon Hospital, 13 Samjungja-ro, Changwon, 51472 Republic of Korea; 2 Department of Radiation Oncology, Seoul St. Mary’s Hospital, College of Medicine, The Catholic University of Korea, 222, Banpo-daero, Seocho-gu, Seoul, South Korea; 3 Institute of Health Science, Gyeongsang National University, Jinju, Republic of Korea

**Keywords:** Inflammatory breast cancer, Palliation, Hypofractionated radiotherapy

## Abstract

**Background:**

Incurable inflammatory breast cancer (IBC) patients occasionally suffer from general symptoms such as breast pain, bleeding, ulceration, and discharge, and thus require palliative radiotherapy (RT). Hypofractionated RT has many advantages in palliative settings, but very few studies on IBC have been conducted. This study was conducted to evaluate the effects of hypofractionated RT on symptomatic IBC patients.

**Methods:**

Twenty-two patients with IBC who underwent hypofractionated palliative RT between 2010 and 2016 were retrospectively analyzed. RT was performed at a total dose of 42.5–55 Gy with 2.5–3 Gy per fraction. The treatment effects were evaluated with respect to symptom improvement, tumor response, and treatment-related toxicity.

**Results:**

The main symptoms that the patients complained of before RT were pain, bleeding, and discharge. According to the percentage of symptom relief compared with pre-RT symptoms, the number of patients with < 30, 30–70%, and ≥ 70% were 2 (9.1%), 7 (31.8%), and 13 (59.1%), respectively. Eighteen (81.8%) patients showed tumor response. No patient experienced grade 3 or higher acute or chronic toxicity during a median follow-up period of 13 months. In univariate analysis, symptom type was a significant factor for predicting the degree of symptom relief. Meanwhile, RT field and C-reactive protein increase were significant factors for predicting the incidence of radiation-induced skin toxicity.

**Conclusions:**

Hypofractionated RT could safely and effectively relieve symptoms among incurable symptomatic IBC patients.

## Background

Inflammatory breast cancer (IBC) is a rare disease that accounts for 0.5–2% of all invasive breast cancers [1]. The standard treatment for non-metastatic IBC is multimodality therapy including neoadjuvant chemotherapy followed by mastectomy and post-mastectomy radiotherapy (RT) [2–4]. However, such aggressive multimodality approach is limited in patients with poor performance status or in approximately 30% of patients with metastatic disease at diagnosis [1]. In such patients, palliative RT is occasionally warranted to control typical symptoms such as breast pain, bleeding, ulceration, and discharge [5, 6].

IBC is known for having more radio-resistance than non-IBC; hence, some studies have suggested more aggressive radiation strategies such as increases in the total radiation dose or changes in the fractionation schedules [7, 8]. In practice, several studies have performed dose escalation with accelerated hyperfractionation schedules in IBC, and these studies have reported an improvement in locoregional control [3, 7, 9, 10]. However, this strategy of treating more than twice per day was limited by loading of the treatment machine and accessibility of patients to a hospital. Another altered fractionation schedule is hypofractionation; hypofractionated RT delivers a daily dose of > 2 Gy per fraction and can not only provide good tumor control but also shorten the total treatment period, thereby improving patient convenience and reducing medical expenses [11–13]. However, there have been very few studies on hypofractionated RT for IBC treatment. This might be because the breast skin is affected by inflammatory changes and appears vulnerable to radiation; hence, to date, concerns such as severe toxicities caused by hypofractionated RT conducted by administering large daily doses and involvement of a short overall treatment period remain.

Therefore, it is necessary to analyze the effects of hypofractionated RT on IBC. Here, we retrospectively analyzed the treatment efficacy and toxicities of incurable and symptomatic IBC patients who underwent hypofractionated RT.

## Methods

### Patient selection

Patients who underwent palliative-intent hypofractionated RT for symptomatic IBC between January 2010 and February 2016 at Seoul St. Mary’s Hospital, Seoul, were selected for this analysis. Patients with any of the following conditions were included in this study: 1) presence of clinicopathologically proven IBC based on the 8th edition American Joint Committee on Cancer TNM staging system [14, 15], 2) judged medically or surgically incurable as judged by a multidisciplinary team, and 3) presence of symptoms such as breast pain, bleeding, ulceration, or discharge requiring urgent symptom palliation. Patients who were initially diagnosed with a metastatic or refractory disease after the initial chemotherapy or hormone therapy were included in this analysis. In contrast, patients with any of the following conditions were excluded: 1) previous history of breast surgery or RT, 2) refusal of RT for personal reasons, and 3) male breast cancer. Thus, a total of 22 IBC patients were eligible for this analysis. Electronic medical records, RT plans, and work-up images were reviewed to analyze treatment effects.

This study was a retrospective, with no informed consent from individual patients, but was performed in accordance with relevant guidelines; the study protocol was approved by the intra-institutional ethics committee of the Seoul St. Mary’s Hospital (assigned number: KC15RISI0260).

### Hypofractionated RT

All patients were immobilized with vacuum bags in the arm-up position and computed tomography (CT) scans of their breast were taken. These CT images were imported into Pinnacle treatment planning system, Version 9.1 (Philips Radiation Oncology Systems, Fitchburg, WI, USA). In RT planning, whole breast and skin were not routinely targeted. The principle of target delineation was as follows. The gross lesion responsible for breast symptoms was defined as the gross tumor volume. This volume sometimes included the adjacent metastatic axillary lymph nodes if it was directly connected to the primary tumor. The clinical target volume was not delineated because treatment was delivered with palliative intent. Subsequently, a volumetric margin of 1.0 cm was applied to create the planning target volume (PTV). If PTV overlapped with the lung or heart, it was manually modified to exclude these organs. Basically, RT plan was designed using 6 MV tangential fields with wedges. Bolus was applied every other day to cover PTV. Two patients for whom the primary mass extended up to the supraclavicular area were also treated with a single anterior oblique photon field. All patients were prescribed a total dose of 42.5–55 Gy with 2.5–3 Gy per fraction, once per day, 5 days a week, for 3–5.5 weeks. In these modest hypofractionated regimens, the total dose was 69.1–89.4 Gy biologically effective dose (BED), which was calculated assuming an alpha/beta ratio of four for breast cancer [16]. The RT plan was normalized such that 100% of PTV received more than 95% of the prescribed dose. The ipsilateral mean lung dose was limited to ≤20 Gy with V_20Gy_ of < 30%. The heart was limited to V_35Gy_ of < 30% (V_xGy_: the percentage of the organ volume receiving x Gy or more).

### Follow-up and statistical analysis

Before starting RT, all patient symptoms were recorded on a 0–10 numeric rating scale. During the course of RT, patients were evaluated weekly to assess the degree of symptom relief and treatment-related toxicities. After the end of the RT, patients were generally followed-up at 1 week, then every 2 months for the first year, and then every 6 months for the next 3 years. Symptom relief was quantified by the patient as a percentage of symptom relief compared to their baseline. The degree of symptom relief was divided into three categories by modifying Agarwal et al. study [17] as percentage relief < 30%, percentage relief 30–70%, and percentage relief > 70%. Toxicity was evaluated by the National Cancer Institute’s Common Terminology Criteria for Adverse Events (CTCAE) version 4.03. Serum C-reactive protein (CRP) assay was performed before RT, every week during the RT, and then upon follow-up to estimate the degree of skin irritation through increased systemic inflammation marker [18, 19]. CT was performed at 2 months after RT, and every 3 months afterward for treatment response evaluation. The response was evaluated by the Response Evaluation Criteria in Solid Tumors (RECIST) criteria. Overall survival (OS) duration was defined as the period from the end date of RT to the date of death for any reason. OS analysis was performed using the Kaplan–Meier method. The relations between patient, tumor, and RT characteristics with treatment outcomes (symptom relief and toxicity) were evaluated by univariate analysis using logistic regression. Symptom relief was analyzed based on 1-month results after the end of RT when the most clinically symptomatic improvement is expected. Treatment-related toxicity was analyzed based on all adverse events occurring during the follow-up period. All analyses were performed using the SPSS program (Version 21.0; SPSS, Inc., Chicago, IL, USA). *P* values < 0.05 were considered statistically significant.

## Results

### Patient characteristics

A total of 22 patients were included in this study. Table 1 provides a summary of the patients’ characteristics. Their median age at the time of RT was 66 years (range: 39–90 years). The Eastern Cooperative Oncology Group performance score for most (90.9%) of these patients was 0 or 1. According to the 8th TNM staging system, the number of patients with N0, N1, N2, and N3 was 1 (4.5%), 1 (4.5%), 11 (50%), and 9 (40.9%), respectively. Regarding M stage, 10 (45.5%) patients were in the M0 stage, while 12 (54.5%) were in the M1 stage. Nine (40.9%) patients had received chemotherapy or hormone therapy before RT, but the disease continued to progress. Regarding RT technique, the prescribed dose was < 80 Gy (BED, alpha/beta ratio = 4) for 31.8% (7/22) of patients and ≥ 80 Gy (BED, alpha/beta ratio = 4) for 68.2% (15/22) of the patients.

**Table 1:** Patient characteristics

Characteristic		Number of patients
Age (years)		Median: 66 (range, 39–90)
ECOG PS	0 or 1	20 (90.9%)
	2	2 (9.1%)
Pathology	IDC	20 (90.9%)
	IMC	2 (9.1%)
N stage	0 or 1	2 (9.1%)
	2 or 3	20 (90.9%)
M stage	0	10 (45.5%)
	1	12 (54.5%)
Previous CTx or HTx history	Yes	9 (40.9%)
	No	13 (59.1%)
Prescribed dose (BED)	70–80 Gy	7 (31.8%)
	≥80 Gy	15 (68.2%)

*ECOG* Eastern Cooperative Oncology Group, *PS* performance status, *IDC* invasive ductal carcinoma, *IMC* invasive medullary carcinoma, *CTx* chemotherapy, *HTx* hormone therapy, *BED* biologically effective dose

After approximately 1 month of RT, palliative chemotherapy or hormone therapy was performed in 77.3% (17/22) of patients. The median follow-up duration was 13.9 months (range, 1.1–81.2 months), and six patients were alive at the end of the follow-up period. The median OS was 17.3 months, and 1-year and 2-year OS rates were 62.8 and 34.5%, respectively.

### Symptom relief and tumor response

The main symptoms requiring palliative RT were pain (13 patients, 59.1%), bleeding (4 patients, 18.2%), and discharge (13 patients, 59.1%). Eight patients had two or more combination of symptoms. Stratification for symptom relief (< 30, 30–70%, and ≥ 70%) was performed according to the percentage of symptom relief in comparison with the initial symptoms, and the results are listed in Table 2. In total, the number of patients with < 30, 30–70%, and ≥ 70% symptom relief were 2 (9.1%), 7 (31.8%), and 13 (59.1%), respectively. Among the main symptoms, pain relief tended to be more prominent than decrease in bleeding or discharge. Two patients who had symptom relief of < 30% after RT subsequently received palliative chemotherapy, but the symptoms continued to deteriorate rapidly and the patients died shortly afterward. After evaluating the response according to RECIST criteria, 18 (81.8%) patients showed a partial response, three (13.6%) patients showed stable disease, and the remaining one (4.5%) patient showed progressive disease during the follow-up period. Table 3 shows the univariate analysis of patient characteristics on the degree of symptom relief. In the univariate analysis, symptom type (*p* = 0.02) was a significant clinical factor for predicting the degree of symptom relief. Patients with a single symptom had significantly better symptom relief than those with overlapping symptoms. In addition, although not statistically significant, symptom relief tended to be better in patients who received a high radiation dose (BED ≥80 Gy, a/b ratio = 4, *p* = 0.06).

**Table 2:** Degree of symptom relief at 1 week after the end of radiotherapy

	Degree of symptom relief compared to baseline		
	< 30%	30–70%	≥70%
Pain (*n* = 13)	1 (7.7%)	3 (23.1%)	9 (69.2%)
Bleeding (*n* = 4)	0	3 (75%)	1 (25%)
Discharge (*n* = 13)	2 (15.4%)	7 (53.8%)	4 (30.8%)
Total (*n* = 22)	2 (9.1%)	7 (31.8%)	13 (59.1%)

**Table 3:** Univariate analysis of the degree of symptom relief

Variables	HR	95% CI	*p* value
Pathology (IDC vs. IMC)	0.36	0.06–2.23	0.27
Previous CTx or HTx history (Yes vs. No)	0.68	0.14–3.25	0.63
Prescription dose (< 80 Gy_4_ vs. ≥80 Gy_4_)	0.70	0.05–5.12	0.06
Symptom type (single vs. combination)	3.57	1.87–49.08	0.02
Tumor response (PR vs. SD + PD)	0.16	0.01–1.84	0.14

*HR* hazard ratio, *CI* confidential interval, *IDC* invasive ductal carcinoma, *IMC* invasive medullary carcinoma, *CTx* chemotherapy, *HTx* hormone therapy, *Gy*_*4*_ biologically effective dose of alpha/beta ratio 4, *PR* partial response, *SD* stable disease, *PD* progressive disease

### Toxicity

**Fig. 1:**
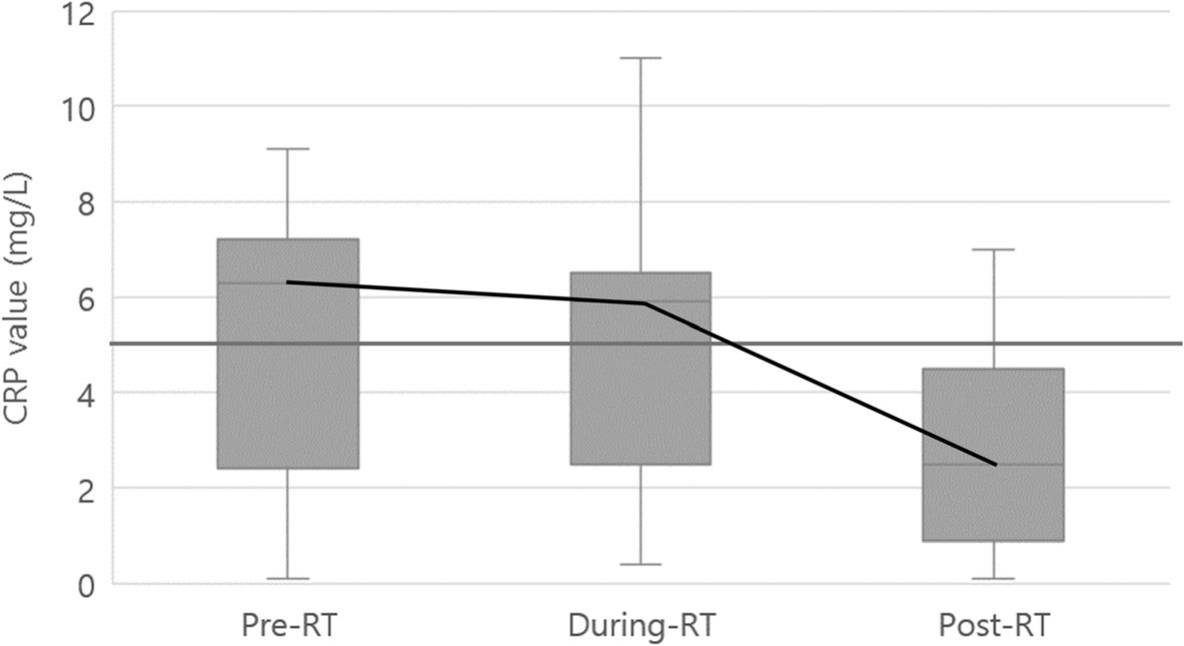
Box-and-whisker plot of C-reactive protein level according to time based on radiotherapy. The straight gray line is the normal value (0–5 mg/L) drawn for reference, and the black trend line shows the change in the median value. CRP, C-reactive protein; RT, radiotherapy

Treatment was well-tolerated. Based on CTCAE criteria, acute complications during and shortly after RT were grade 1 skin dermatitis (faint erythema and dry desquamation) in six patients (27.3%) and grade 2 skin dermatitis (moist desquamation) in 16 patients (72.7%). No patient developed grade 3 or higher skin toxicity and none died from treatment-related toxicity. Chronic complications of radiation, severe chest wall fibrosis, rib fracture, arm edema, and symptomatic pneumonitis were not observed in any patient. These side effects in patients were improved by appropriate supportive care such as frequent dressing, application of topical agents, and collaboration with a dermatologist when necessary. To quantitatively analyze the degree of skin dermatitis, changes in CRP values before, during, and after the RT period were measured. The normal CRP value was 0–5 mg/L. In four patients (18.2%), the CRP level gradually increased during the RT period and fell to the normal range 1 month after RT. The median CRP values before (1 week before RT), during (2 weeks during RT), and after the RT period (1 month after RT) were 6.3, 5.9, and 2.5 mg/L, respectively. Figure 1 shows the change in CRP median values through box-and-whisker plots. Table 4 shows the univariate analysis of patient characteristics on skin toxicity according to CTCAE. In the univariate analysis, the RT field (*p* = 0.04) and CRP increase during the RT period (*p* = 0.03) were significant factors for predicting the incidence of radiation-induced skin toxicity.

**Table 4:** Univariate analysis of skin toxicity according to CTCAE

Variables	HR	95% CI	*p* value
Age (< 65 years vs. ≥65 years)	0.76	0.04–3.21	0.49
Previous CTx or HTx history (Yes vs. No)	1.21	0.43–4.24	0.55
RT fraction size (2.5 Gy vs. > 2.5 Gy)	0.89	0.01–2.05	0.18
Prescription dose (< 80 Gy_4_ vs. ≥80 Gy_4_)	0.92	0.31–6.17	0.25
RT field (breast mass vs. breast + SCL)	0.86	0.01–5.45	0.04
CRP increase during the RT period (Yes vs. No)	2.52	1.01–8.92	0.03

*HR* hazard ratio, *CI* confidential interval, *CTx* chemotherapy, *HTx* hormone therapy, *RT* radiotherapy, *Gy*_*4*_ biologically effective dose of alpha/beta ratio 4, *SCL* supraclaviculr lymph node, *CRP* C-reactive protein

## Discussion

We retrospectively analyzed 22 patients who received hypofractionated RT for symptomatic IBC. Most patients showed symptom relief with acceptable skin toxicity. Although OS was much poorer than that of patients who underwent curative-intent standard multimodality treatment, 81.8% of patients showed a tumor response within the follow-up period.

It is uncommon to encounter unresectable breast cancer patients in this era when screening tools have been developed and neoadjuvant chemotherapy can be performed. Despite not being tumor resected, RT still plays an important role in women suffering from ulceration, bleeding, or pain in locally advanced and recurrent breast cancer [20, 21]. Although there are no clear guidelines for the most appropriate palliative RT dose and fractionation schedules, tumor control tends to increase with increasing total radiation dose [22]. In addition, hypofractionated schedules were often used in palliative RT to improve patient inconvenience by reducing treatment duration [23–25]. Based on this knowledge, hypofractionated palliative RT of 42.5–55 Gy with 2.5–3 Gy per fraction was performed in our study to increase the RT effect. This schedule was similar to the dose schedules used in hypofractionated whole breast RT studies for adjuvant aim after breast conservation surgery, and long-term results of these studies have revealed that this schedule has a safe outcome [13, 26, 27].

To date, no study has focused on hypofractionated palliative RT of IBC patients; hence, it is difficult to undertake a direct comparison of the palliation effect. However, indirect comparisons with existing non-IBC studies are possible. Richard et al. [5] reported the effect of palliative RT on 13 locally advanced breast cancer patients with skin ulceration. The median dose was 27.54 Gy in 11 fractions, and 46.2% of patients showed clinical improvement. Only doses that exceed 30 Gy were found to be effect in patients. Nakamura et al. [28] performed a prospective study to evaluate the effectiveness of various palliative RT regimes for 21 patients with breast cancer including skin invasion; most of their patients received 36 Gy in 12 fractions, but some patients received a relatively high-dose regimen such as 50 Gy in 20 fractions or 60 Gy in 30 fractions. In a majority of the patients in that study, bleeding, discharge, and offensive odor reduced and the quality of life score improved. However, they suggested that dose fractionation optimization is necessary because symptoms tended to re-progress after approximately 6 months. In our study, approximately 60% of patients reported ≥70% symptom improvement in comparison with pre-RT symptoms, and these improvements were marked by a single symptomatic pain. Although not statistically significant, symptomatic improvement was detected at dosed greater than BED 80 Gy (a/b ratio = 4).

RT-related toxicity was influenced by both fraction size and a wide variety of factors. Bristol et al. reported 10–20% grade 3 or higher treatment-related toxicity in IBC patients receiving curative-intent aggressive multimodality treatment [3]. In contrast, Whelan et al. reported < 3% toxicity of grade 3 or higher in patients with early breast cancer who underwent hypofractionated RT following breast-conserving surgery [13]. In our study, neither late toxicity within the follow-up period nor acute toxicity of grade 3 or higher was observed. The difference in toxicity between these studies was considered to be due to the RT field, which was considered to be a significant factor affecting toxicity and extent of surgery in our study. We believe that the toxicity was low in our study because surgery was not performed in all the patients, and irradiation was limited to the tumor mass and did not frequently include the regional lymphatic areas. Our study also revealed that the CRP level did not increase with RT in most patients. Of course, the CRP level not only reflects inflammation of the skin but also of the whole body; the CRP level can change for various reasons including bacterial superinfection and cancer progression. However, some studies have reported a causal relationship between skin dermatitis and CRP levels [19, 29]. To minimize CRP level variation, we performed repeated measurements of CRP levels at 1-week intervals. In addition, we examined the use of antibiotics to influence the CRP level and confirmed that no antibiotic was used in any patients during the RT period. Patients with increased CRP levels were more likely to develop skin toxicity; hence, active supportive care can be applied according to CRP level changes.

This study has several limitations. The first was the retrospective nature of the study, which may have resulted in heterogeneous patient characteristics particularly among 40.9% of patients who had received previous chemotherapy or hormone therapy, and the second was the relatively small number of cancer patients. However, our study is meaningful in that it focused on patients with IBC as well as on the effects of hypofractionated palliative RT. In future, further studies with more patients will be required to confirm the effect of hypofractionated palliative RT on the quality of life of patients or on complications seen later during the long-term follow-up. In addition, it is possible to conduct studies to examine the increase in local control and disease-free survival by adding aggressive treatment to patients who respond to hypofractionated RT.

## Conclusions

Our results suggest that hypofractionated RT was relatively effective fo the mono-symptom of breast pain in incurable and symptomatic IBC patients. In addition, this treatment was safe and well tolerated with acceptable toxicity. Further studies to determine an appropriate RT scheme, such as dose fractionation schedule and RT field, are warranted to balance the effects and toxicity.

## Data Availability

Raw data may be available on request from the corresponding author.

## Abbreviations

BED: Biologically effective dose
CRP: C-reactive protein
CT: Computed tomography
CTCAE: Common Terminology Criteria for Adverse Events
IBC: Inflammatory breast cancer
OS: Overall survival
PTV: Planning target volume
RECIST: Response Evaluation Criteria In Solid Tumors
RT: Radiotherapy
